# Acknowledgment to the Reviewers of *Membranes* in 2022

**DOI:** 10.3390/membranes13020122

**Published:** 2023-01-18

**Authors:** Membranes Editorial Office

**Affiliations:** MDPI AG, St. Alban-Anlage 66, 4052 Basel, Switzerland

High-quality academic publishing is built on rigorous peer review. *Membranes* was able to uphold its high standards for published papers due to the outstanding efforts of our reviewers. Thanks to the efforts of our reviewers in 2022, the median time to first decision was 13 days and the median time to publication was 32 days. Regardless of whether the articles they examined were ultimately published, the editors would like to express their appreciation and thank the following reviewers for the time and dedication that they have shown *Membranes*:

Abbas, IbrahimLow, NicholasAbdala, AhmedLowdon, Joseph W.Abdallah, HebaLoza, NataliaAbdel-Fatah, Mohamed E. A.Lozano, Angel E.Abdel-Hamid, Shereen M. S.Lu, ChiyuAbdul-Hamid, Muhammad AzmiLu, Hiep ThuanAberoumand, SadeghLu, PengAbidin, Muhammad Nidzhom ZainolLu, XiaofeiAboraia, A. M.Łukasz Konefał, RafałAbushaban, AlmotasembellahLunavat, TaralAcero, Juan L.Lundin, Sean-Thomas B.Adelodun, BashirLuo, BirongAdeniji, Abiodun OlagokeLuo, FabaoAdi, Vincentius Surya KurniaLuo, JianchengAfsar, Noor UlLuo, ShuangjiangAgostini, AllesandroLutic, DoinaAgrahari, VibhutiMa, CanghaiAgriesti, FrancescaMa, Chih-MingAhmad, Mohd ZamidiMa, HongyangAhmed, SafeerMa, ShengcunAhn, Jun KiMa, WenjingAkhtar, Faheem HassanMacedonio, FrancescaAkram, NadiaMachaca, KhaledAkyuz, EnesMadhuranthakam, Chandra Mouli RAl Munsur, Abu ZafarMafalda, SantosAlam, ManawwerMagomedov, Kurban E.Alayande, Abayomi BabatundeMahdipour, MahdiAlfano, BrigidaMahesh, Dhavale VishalAlfuraiji, MustafaMahmood, Raid AhmedAlgadi, HassanMahmoodi, Niyaz MohammadAlghoraibi, IbrahimMahmoud, Ahmed AbdulhamidAlhuyi-Nazari, MohammadMahmoudi, ShahramAli, ImranMaidaniuc, AndreeaAli, IsratMaidhof, RobertAli, Syed AzmalMakisha, NikolayAli, ZeeshanMaleki, RezaAl-jubouri, SamaMandelli, DavideAlmásy, LászlóMangiarotti, AgustínAlmeida, InesMangindaan, DaveAl-Mubarak, Bashayer R.Mangino, GiorgioAl-Obaidi, Mudhar A.Maniewska, JadwigaAl-Sabi, AhmedMannella, Carmen A.Alsalhy, QusayManohar, MurliAl-shaeli, MuayadManouras, TheodorosAlshahrani, AhmedMansourizadeh, AmirAlshami, Ali S.Mansourpanah, YaghoubAlsohaimi, IbrahimManzar, Mohammad SaoodAltamash, TausifMaqsood, KhuramAlzahrani, FaisalMarangoni, CintiaAl-Zoubi, WailMarcos, Paula M.An, XiaochanMarek, JaromírAnashkin, IvanMariani, PaoloAnastasiadi, Alkmini T.Marin-Gozalbo, AnnaAnnan, EbenezerMarjani, AzamAnnesini, Maria CristinaMarkiewicz, RoksanaAnstine, DylanMarkov, DmitryAnticó, EnriquetaMarpani, FauziahAntipov, AnatolyMarquês, Joaquim Manuel TrigoAntipova, Veronica AlexandraMarques, Marta CoimbraAntoniewska, AgataMarszałek, JoannaAnwar, MumtazMartens, ChloeAnwar, TauseefMarti, JordiAnwer, Md KhalidMartín, CristinaArafat, HassanMartin, SimonArain, Muhammad BalalMartín-Cófreces, Noa B.Aranowski, RobertMartínez-Huitle, Carlos A.Araújo, TiagoMartinho, MarlèneArce, Florencio V., Jr.Martino, MarcoArdelean, IoanMarzban, GorjiArgirusis, ChristosMarzec, MichałArgitis, PanagiotisMasdar, Mohd ShahbudinArinstein, ArkadiiMashentseva, AnastassiaArjunan, AriharanMashhadzadeh, Amin HamedArkas, MichaelMashifana, TebogoArous, MouradMastropietro, Teresa FinaArrigoni, CristinaMateo Fernandez, SaraArsad, Agus BinMateus, MaríliaArunkumar, AbhiramMatsuki, HitoshiAryal, Uma K.Matsumori, NobuakiAshfaq, Mohammad YousafMatsumoto, HidetoshiAshfaq, MuhammadMatsumoto, MichiakiAshtiani, SaeedMatsuura, KazunoriAsif, SairaMatsuura, TakeshiAsimakopoulou, AkriviMatsuzaka, YasunariAskari, Mohammad BagherMatykiewicz, DanutaAtanasov, VladimirMeans, Robert T.Athinarayanan, BalasankarMedina-Gonzalez, YaocihuatlAtkinson, IrinaMeesorn, WorarinAtlaskin, ArtemMehanny, SherifAwasthi, ShikhaMehdi, EbrahimiAyala, Juan A.Mehmood, KhalidAydoğdu, AyçaMehrazi, ShirinAyyavoo, JayalakshmiMehrotra, Gopal KrishnaAzuma, TakashiMei, GiampieroBabar, MuhammadMei, HaoBabaRao, RavichandarMei, YingBabilas, DorotaMeleleo, Daniela AddolorataBadawi, Ahmed K.Melin, FredericBadmalia, Maulik D.Melnikov, StanislavBae, Byung-ChanMelnikova, GalinaBai, ShuoMelnyk, InnaBaican, Mihaela CristinaMeloni, BrunoBaig, Mirza WasifMendes, RicardoBaig, NadeemMendoza-Roca, José AntonioBaik, Ku YounMenéndez, MiguelBajales, NoeliaMeng, BoBakalár, TomášMensitieri, GiuseppeBaklanov, Mikhail R.Merkel, ArthurBalan, AdrianaMerkulov, Daniela ŠojićBallone, PietroMerlin, SamBalogh, György TiborMescola, AndreaBalta, StefanMestdagh, HélèneBan, YujieMeto, AidaBanchero, MauroMielewczyk-Gryn, AleksandraBanerjee, SayanMihály, AndrásBankala, KrishnarjunaMikheev, IvanBaptista, RosaMikhelson, KonstantinBarbosa, AmiltonMiklavčič, DamijanBarik, BapunMilcovich, GesmiBarman, JiteshMilićević, AleksandarBarragán, V. MariaMilichovský, MiloslavBarros, Kayo SantanaMirabilii, SimoneBasheer Ahamed, M.Mirfendereski, Seyed MojtabaBashyal, SantoshMirzaeei, ShahlaBasiak, EwelinaMishev, KirilBassam, El-EswedMishra, KuldeepBasyigit, BulentMishra, MonalisaBatista, Karla A.Mitko, KrzysztofBatko, KorneliaMitov, MarioBattegazzore, DanieleMitu, BogdanaBayer, IlkerMiyamoto, ManabuBeard, MatthewMizuno, ToshihisaBella, FedericoModestov, Alexander D.Bellanger, GilbertModi, AkshayBello-López, Miguel ÁngelMohamed, Hamdy F. M.Bender, JohannesMohamed, Mohamed A.Benito, José ManuelMohammadi Nafchi, AbdorrezaBento, HeitorMohammed, ShabinBera, Oskar J.Mohd Mokhtar, NadzirahBerezhnov, AlexanderMohy-Eldin, Mohamed S.Bernacchi, SerenaMokhena, Teboho ClementBernardo, GabrielMokhtar, MohamedBernareggi, AnnalisaMolavi, HosseinBertocchi, MichaelMolla, Md. Ashraful IslamBeszédes, SándorMona, ArefpourBeutner, GiselaMonje-Galvan, VivianaBhalani, DixitMonsalve-Bravo, Gloria M.Bhandari, SubhenduMontemurro, DomenicoBhat, SanthoshkumarMontenegro, ConceiçãoBhattacharjee, ArnabMoon, Seung-HyeonBhattacharjya, SurajitMoon, Su-YoungBhole, KiranMoore, Robert B.Bhunia, PrasenjitMoradian, MiladBian, ChaoMorar, AdrianaBian, HuiyangMorikawa, KyojiroBielan, ZuzannaMorishita, HideakiBilad, Muhammad RoilMorita, MasamuneBildyukevich, AlexanderMortalò, CeciliaBin Abd Aziz, Mohd HaiqalMortas, MustafaBita, BogdanMotegi, ToshinoriBizon, KatarzynaMousavi, Seyed BorhanBizzarro, SergioMousavi, Seyyed MojtabaBlandin, GaetanMoutloali, RichardBlázquez, ManuelMsimbira, LeviniBlock, StephanMsomi, PhumlaniBobacka, JohanMu, XuanBobrova, LudmillaMubashir, MuhammadBochkov, ValeryMuhammad Imran, KhanBoddula, RajenderMuller, GerhardBoguang, YangMulligan, Catherine N.Bohinc, KlemenMumtaz, SohailBoldyrev, Ivan A.Munir, Muhammad UsmanBonucci, AlessioMurshid, NimerBorisov, IlyaMurtomäki, LasseBorrega, MarcMusio, CarloBortolus, MarcoNa, YoungseungBortot Coelho, Fabrício EduardoNagarale, Rajaram KrishnaBouquillon, SandrineNaguib, Ibrahim A.Bouropoulos, NikolaosNagy, EndreBouyer, DenisNagy, NorbertBrauchi, SebastianNagy, TiborBravo, Susana B.Nah, JihoonBreite, DanielNaik, PranjaliBrinkmann, TorstenNaito, AkiraBrioni, MatteoNakhjiri, Ali TaghvaieBrouzgou, AngelikiNan, XiaolinBrucale, MarcoNanan, SuwatBruce, Lesley J.Napiórkowska, MariolaBrunklaus, GuntherNarasipuram, Rajanand PatnaikBruno, RosariaNarciso, JavierBryjak, MarekNasir, RizwanBrzhezinskaya, MariaNasr, MahmoudBubola, MarijanNasrollahzadeh, MahmoudBuchsbaum, Steven F.Natale, PaoloBudaeva, Vera V.Naumovich, YevgeniyBuica, George-OctavianNaumowicz, MonikaBuiu, OctavianNawab, YasirBulgariu, LauraNayak, Jagdeep KumarBułkowska, Habil. KatarzynaNayak, VigneshBuonomenna, Maria GiovannaNechifor, GheorgheBuraidah, MohdNemudry, AlexanderBuratta, SandraNesci, SalvatoreBurducea, MarianNesterenko, AlexeyBurghardt, KyleNesterkina, MariiaBurgo, Thiago Augusto LimaNevárez-Moorillón, Guadalupe VirginiaBurlakov, VictorNg, Law YongBusquets, Maria AntòniaNguyen, Duc-NamBustos-Terrones, YanethNieken, UlrichCaballero, AlvaroNikolay, SheldeshovCabezas, RenéNikolic, Maria VesnaCabukusta, BirolNikolova, DimitrinkaCai, YuhangNinan, NeethuCairrão, ElisaNing, HanCakmak, HulyaNitodas, Stephanos F.Calabrò, Paolo S.Njoya, MahometCalvo, José IgnacioNocita, DavideCamacho, Lucy M.Noël, SébastienCambedouzou, JulienNombona, NolwaziCamblor, Miguel A.Nomura, MikihiroCampa, Carlo CosimoNorrrahim, Mohd Nor FaizCampardelli, RobertaNovickij, JurijCañadas, OlgaNunna, Bharath BabuCancelliere, RoccoNypelo, TiinaCandan, ZekiObacz, JoannaCao, BingOchoa, Nelio ArielCao, HongbinOh, Tae-SikCao, WenxinOhayon, RogerCao, YifengOhya, SusumuCapezza, AntonioOjha, Gunendra PrasadCaprarescu, SimonaOksuz, Aysegul UygunCaputo, GregoryOla, Antonius R. B.Carabineiro, SóniaOliva, RosarioCarraro, MichelaOllila, SamuliCarrasquel, WillyOnac, CananCarvalho, Marta S.Onishchenko, Natalia R. O.Casado-Coterillo, ClaraOnukwufor, John O.Castillo, RolandoOo, AdrianCastro-Dominguez, BernardoOpalkova Siskova, AlenaCatro-Muñoz, RobertoOrtega, BartolomeCavaco, Marco C.Ortelli, SimonaCavallari, Marco RobertoOsakoo, NattawutCentonze, DiegoOshiro, João AugustoCerrillo, MiriamOsinkin, Denis A.Cerro-Prada, ElenaOsman, NafisahChae, So-RyongOstolaza, HelenaChakraborty, ChanchalOtosu, TakuhiroChakraborty, SudipOuYang, BoChalmpes, NikolaosOuyang, Xiao-kunChan, Jerry C. C.Ozcelik, AdemChandel, Anuj KumarOzuna, CesarChang, Tong-bouPaganin, Valdecir AntonioChatterjee, SouravPagliero, MarcelloChatterjee, UmaPagot, GioeleChaube, Bal KrishnaPahuja, MohitChauhan, Manvendra SinghPalacio, LauraChavan, PrasadPalazzolo, LucaChawla, PoojaPalazzolo, StefanoChe, QuantongPalma, Paulo J.Chechik, VictorPanchal, HiteshChen, BeibeiPanda, Pradeep KumarChen, Chin-WenPandey, Abhay K.Chen, GuiningPandíțh, AnúpChen, GuoqiangPankratov, VladimirChen, LianjunPant, BishweshwarChen, LukePaolone, AnnalisaChen, MingliangPapp, FerencChen, Shiao-ShingPapurello, DavideChen, Szu-yuanParadowska-Stolarz, AnnaChen, WeiPáramo-García, UlisesChen, XiaoyiParia, SarbaranjanChen, YaoyaoParimelazhagan, VairavelChen, YianPark, ChanhyukChen, ZhaoboPark, Chan-WooChen, ZheyuanPark, Chul-hoChen, ZhimingPark, Jin YongChen, ZhongjianPark, Jin-SooChen, ZhuoPark, Jun-GyuCheng, ChaoPark, Sang-HeeCheng, HuanyuPark, Seong-JikCheng, JohnPark, Seung-minCheng, YouliangParmar, Malvinder S.Cheng, YuweiParra, MargaritaCheraghian, GoshtaspParshina, AnnaChernyshev, VasiliyParthiban, AnburajanCheshmedzhieva, DianaParvasi, PayamChetty, RaghuramPârvu, Alina ElenaChhetri, ManjeetPasechnik, SergeyChiabrando, Gustavo AlbertoPasichnyk, MariiaChien, Andrew C.Pasini, DarioChinarro, EvaPastor, Ferenc T.Chiriacò, Maria SerenaPastore, AndreaChlanda, PetrPatel, SanjayCho, Cheong-WeonPathak, DineshCho, KyungjinPati, SiddharthaCho, YoungHoonPati, SubhasisChoi, Angelo EarvinPatki, NeilChoi, Bum-RakPatsios, Sotiris I.Choi, Nag-ChoulPaul, DennisChoi, WansukPavlov, EvgenyChoi, Young-WooPavlović, SlađanChong, Parskson Lee GauPawlowski, SylwinChong, Woon ChanPedone, Emilia M.Chopra, AditiPei, HaiyanChowdhury, AbhishekPeixoto, FranciscoChu, HuaqiangPereira, Carlos DiasChu, XiangpingPérez, Alan D.Chuah, Chong YangPerez, EdsonChuah, Yew KhoyPérez, MaríaChugunov, Anton O.Perez-Maqueda, LuisChung, Neal Tai-ShungPérez-Puyana, Víctor ManuelChung, Ren-JeiPergher, Sibele B.C.Chung, Sang KugPeriakaruppan, RajivChung, Sheng-HengPerinelli, Diego RomanoChwojnowski, AndrzejPervez, Md. NahidCiesielczyk, FilipPervov, Alexei G.Coelhoso, IsabelPeter, RobertCofelice, MartinaPeters, JudithCojocaru, CorneliuPetriev, IliyaColdea, Teodora EmiliaPetrov, MikhailColeman, MatthewPetukhov, AntonColombo, MartinaPetukhov, Dmitriy I.Corat, Evaldo JoséPhaechamud, ThawatchaiCourjaret, RaphaelPiano, MartinaCowan, Matthew GreigPiątkiewicz, WojciechCristian, PetcuPiazza, FabriceCristina, Romeo TheodorPichugov, RomanCuccovia, Iolanda MideaPiekutin, JaninaCui, JunkuiPiermarini, PeterCzechowska, JoannaPiesik, DariuszDąbrowski, WojciechPietrucha-Urbanik, KatarzynaDad, AliPiiper, AlbrechtDaigle, Jean-ChristophePilipovic, AnaDangwal, Shailesh SinghPinto da Silva, LuísDanilenko, MichaelPinto, LuísDantas, AdrianaPiosik, EmiliaDao, Van-DuongPiper, AndrewDas, RaselPísařík, PetrDas, RatulPitt, SamanthaDash, Santanu KumarPlata-Rueda, AngelicaDassanayake, Rohan S.Platta, HaraldDavidović, SlađanaPlisko, Tatiana V.Davis, FredPolett Gurrola, MayraDavletbaeva, Ilsiya M.Polisetti, VeerababuDe Bruijn, Johannes P. F.Pollice, AlfieriDe Guzman, Manuel ReyesPonczek, Michał BłazejDe Lima, Bruno SanchesPonnuchamy, VeerapandianDe Luca, GiorgioPontarin, GiovannaDe Marcos-Lousa, CarinePontes, FredericoDe Oliveira, Helinando PequenoPontié, MaximeDe Oliveira, Ivan PiresPorozhnyy, MikhailDębowski, MarcinPorro, ChiaraDeemer, EvaPotiyaraj, PranutDeivasigamani, Ranjith KumarPottosin, IgorDel Carratore, RenataPoveda, Jose AntonioDel Castillo, Roxana MitzayéPoyatos, JoséDemakov, PavelPozzobon, VictorDemana, PatrickPradanos, PedroDe-Miguel, Francisco F.Pramanik, Atinden Otter, Wouter K.Prasad, BabulDeng, LiyuanPreobrazhenskiy, AndreyDerbal, KerroumPresley, JohnDerler, IsabellaPrihandana, Gunawan SetiaDeshpande, GauraviPriyadarshi, RuchirDessie, YilkalPrudovsky, IgorDhakshinamoorthy, AmarajothiPrzybyło, MagdalenaDhar, GouravPtaszek, AnnaDheyab, Mohammed AliPucek, AgataDi Bella, SantinaPugazhenthi, GopalDi Girolamo, RoccoPuitel, Adrian CǎtǎlinDi Lella, SantiagoPulyalina, AlexandraDi Martino, GiuseppePunia, SnehDi Profio, GianlucaPuragliesi, RiccardoDietrich, ThomasPurwidyantri, AgnesDing, DongyanPuscaselu, RoxanaDisalvo, AnibalPushkarev, ArtemDivino-Filho, JosèPustovalova, AllaDo, Khac-UanPutz, Ana MariaDoan, HoanPylaev, TimofeyDobrzańska, JoannaQadir, DanialDobrzyńska-Inger, AgnieszkaQaisrani, Naeem AkhtarDoğan, Hülya öztürkQazi, Umair YaqubDoi, KentaroQian, ShuoDokollari, AleksanderQiang, YuhaoDoktorova, MilkaQin, WeiDolowy, KrzysztofQin, YuyueDong, ChunhongQu, ZhiguoDong, WeilingQue, WenxiuDong, XiaoboQuijada-Maldonado, EstebanDongare, PratikshaRaceanu, MirceaDordevic, DaniRacovita, RaduDorn, IsabelRadha Shanmugam, NandhineeDorozhkin, Sergey V.Radoor, SabarishDrioli, EnricoRadosavljević, TatjanaDuan, PuRadu, MihaiDucza, EszterRadzymińska-Lenarcik, ElżbietaDudek, GabrielaRaed, Al-JubooriDumitru, FlorinaRafatullah, MohdDunaevskiy, MikhailRaghu, Anjanapura V.Dunyushkina, LiliyaRai, AmbakDuranton, ChristopheRaineri, DavideDutta, SubhasishRajabi, ArminDzyazko, YuliyaRajan, KalavathyEbrahimi, AminRajendran, Ramya LakshmiEdomwonyi-Otu, Lawrence C.Raji, AtchudanEfimova, Svetlana S.Ramakrishnan, SathishÉgée, StéphaneRamalho, Teodorico de CastroEğri, SinanRamapuram, JayEkka, BasantiRamazani, AliEl Hasadi, YousefRambabu, KrishnamoorthyEl Nashar, Rasha MohamedRamesh, ManickamEl-Aassar, MohamedRana, DipakEl-Azazy, MarwaRankin, SteveElboughdiri, NoureddineRao, TejeshwarElcik, HarunRather, Sami-ullahElekhnawy, EngyRätzke, KlausElemans, Johannes A. A. W.Raval, Hiren D.Elewa, Mahmoud M.Ravichandran, SantoshElhenawy, YasserRavishankar, PrashanthEliseeva, TatianaRay, Saikat SinhaEljaddi, TarikRazdan, SidharthElkhenany, HodaRehman, Muhammad MuqeetElma, MuthiaReif, MariaElmarghany, Mohamed R.Ren, GuipingElsaid, KhaledRen, ShanEl-Sayed Nasef, Mohamed MahmoudRestivo, JoãoEl-Shafie, MostafaRetno Ariadi, LusianaEl-Shahat, MahmoudReyes-Gasga, JoséElSherbiny, Ibrahim M.A.Reyes-López, Simón YobannyEnglart, SebastianRezaeigolestani, MohammadrezaErmakova, ElenaRezakazemi, MashallahEscorihuela, JorgeRezk, HegazyEskelin, KatriRhi, Seok-HoEslami, HosseinRibeiro, Viviana PintoEspinosa-Juárez, Josué V.Richert, AgnieszkaEum, KiwonRigalli, Juan PabloEvanghelidis, AlexandruRiswanto, Florentinus Dika OctaEvergren, EmmaRius-Ayra, OriolEzzat, Mohammed OdayRivera Gavidia, LuisFaccio, RicardoRobert, JérômeFahrner, MarcRobles, AngelFan, Huan-JungRodrigues, ArmandaFan, YujiangRodríguez, Francisca A.Farajzadeh, NazliRodríguez-Gómez, Luis E.Faria, MonicaRodríguez-Ortega, Manuel JoséFato, RomanaRohner, PatrikFatyeyeva, KaterynaRojas, AdriánFavvas, Evangelos P.Rokitskaya, Tatyana I.Feng, YingangRona, RobertoFernandes, Renata SalgadoRos, UrisFernández de Labastida, MarcRosa, Luis GuerraFeroz, HasinRossouw, ArnouxFerreira, Rodrigo S.Rostami, AmirFerreira, Rui B.Rostoker, GuyField, RobertRostovtseva, TatianaFierascu, Radu ClaudiuRotaru, AndreiFilice, SimonaRothnie, AliceFilip, PetrRousset, MatthieuFilipe, Rui M.Roux De Balmann, HélèneFilipescu, MihaelaRoy, SagarFilipkowska, UrszulaRóżański, Stanisław AndrzejFilipova, IneseRózsenberszki, TamásFilippov, AnatolyRubakhin, StanislavFisch, AdrianoRubart, MichaelFitter, JörgRubina, Margarita S.Fologea, DanielRuby-Figueroa, RenéFolorunso, OladipoRuffino, BarbaraFontàs, ClaudiaRuiz-García, AlejandroFormoso, PatriziaRusso, FrancescaFrampton, Mark B.Russo, GiacomoFranca, Eduardo F.Russo, PietroFrancesco, Giovanni DeRusso, TeresaFrancis, LijoRusu, Radu-DanFranco, AntoniettaRüther, ThomasFrielinghaus, HenrichRuysschaert, Jean-MarieFriess, KarelRychkov, GrigoriFröhlich, KarolRyi, Shin-KunFu, RongqiangRyu, Yu KyoungFujita, ToyohisaRyzhkov, Ilya I.Fukuda, MakotoSabbah, MohammedFung, Kuan-ZongSachar, Harnoor SinghFuzi, Nursyazwani MohdSachio, TsuchidaG. Gaidin, SergeiSadowska, KamilaGabriela, PaunSadrzadeh, MohtadaGaburjáková, MartaSadykov, VladislavGahruie, Hadi HashemiSaeed, SaqibGaidukovs, SergejsSaeed, Waseem SharafGaikwad, Kirtiraj KundlikSafonov, AlexeyGajewski, PiotrSaha, BidyutGajtko, OlgaSahu, Indra D.Galassi, VanesaSaid, Ahmed S.Gallegos, AlbertoSaifutdinov, BulatGallo, JuanSajjan, Ashok M.Gamba, GerardoSalamango, DanielGan, WeiSalari, MarjanGanesapillai, MaheshSalem, Mohamed Z. M.Gangadaran, PrakashSalem, TamerGao, JieSalinas-Rodriguez, Sergio G.Gao, ShiqiangSalvado, VictoriaGao, YuanSamaras, PetrosGarcia-Bernabé, AbelSamhaber, Wolfgang M.Garcia-Gomez, CelestinoSampaio-Maia, BeneditaGarcía-Márquez, AlfonsoSamsudin, Asep MuhamadGarea, AuroraSanchez-Salvador, Jose LuisGatto, EmanuelaSancho-Vaello, EneaGauden, Piotr A.San-Román, M. FresnedoGavrila, Ana-MihaelaSantambrogio, CarloGbadamosi, AfeezSanthosha, Aggunda L.Gdula-Argasińska, JoannaSantiago, José AntonioGe, LiangSanto, Kolattukudy PouloseGeise, Geoffrey M.Santos, Arnaldo R., Jr.Genduso, GiuseppeSanyal, UdishnuGennaro, RenatoSapalidis, AndreasGeorgin, JordanaSaraswat, VivekGeorgopanos, ProkopiosSargolzaeiaval, YasamanGergely, AttilaSarmento, Maria J.Ghadai, RanjanSarti, Giulio C.Ghalkhani, MasoumehSatilmis, BekirGhasem, Nayef MohamedSato, ChikashiGhosh, DebopamSato, YuyaGhosh, RajaSayed, Ibrahim M.Giacobbo, AlexandreSayyed, SirajGiannoulis, AngelikiSazali, NorazlianieGierszewska, MagdalenaSazonov, OlegGil, ViolettaScalese, SilviaGiliopoulos, DimitriosScarazzato, TatianaGiorno, LidiettaSchneck, EmanuelGlaß, SarahSchneider, DirkGloria, AntonioScholz, CarmenGnani Peer Mohamed, Syed IbrahimSchöning, Michael J.Goda, Emad S.Schönthal, Axel H.Goddard, AlanSchubert, DirkGoh, KunliSchütt, FabianGoldmann, MichelSchweiss, RuedigerGolubeva, TatianaSeabra, GustavoGomez, Miguel AngelSeçkin, TurgayGomez-Coma, LuciaSeddighi, MehdiGomez-Pinedo, UlisesSedlaříková, JanaGoncalves, VivianeSee, Hong HengGondolini, AngelaSegredo-Morales, ElisabetGong, XuzhongSegura Segura, RodrigoGordano, AmaliaSelçuk, HüseyinGorgieva, SelestinaSelektor, Sofiya L.Gosling, MartinSelyutin, Artem A.Gotzias, AnastasiosSemsarilar, MonaGouda, Marwa H.Sengupta, DebarunGowda, Ramanahalli Jayadevamurthy PunithSenju, YosukeGrabska-Zielińska, SylwiaSenthil, RethinamGranado Castro, María DoloresSequeira, César Augusto CorreiaGray, StephenȘerban, Bogdan CătălinGreish, YaserSereda, VladimirGrigoriev, FedorSeruga, PrzemysławGrivas, IoannisSeshimo, MasahiroGrochowicz, MartaSethi, SushantaGroschner, KlausSevastianov, Viktor I.Grumetto, LuciaSgarlata, CarmeloGrushevenko, EvgeniaSgreccia, EmanuelaGuan, KechengShafiquzzaman, MdGuarino, Andrea MariaShah, Aatif AliGuaus, EsterShahid, Muhammad KashifGudipati, ChakravarthyShahzad, AsifGuerrero-Sanchez, CarlosShahzad, Muhammad WakilGuibal, EricShaik, Mohammed RafiGuille, MattShamsuddin, NorazanitaGuillén, ElenaShang, ZhihaoGüler, EnverShanmugavelayutham, GurusamyGunathilake, ChamilaShao, LuGunawan, PoernomoShao, SenlinGuo, DandanSharan, PreethaGuo, FeiSharifian, SeyedmehdiGuo, HeSharma, MahendraGuo, JinSharma, PriyankaGuo, WeijinSharma, Sunil K.Guo, YouzhongSheehan, MadocGuo, ZhanhuShehata, NaderGupta, MeghnaShen, JialongGupta, OindrilaShen, YuxiaGupta, SanjuShevate, RahulGurau, VladimirShevchenko, Vitaliy G.Gurreri, LuigiShibuya, MasafumiGuskov, AlbertShimanouchi, ToshinoriGuzmán, EduardoShintani, YukihiroHaddad, MaryamShishatskiy, SergeyHadi, SutopoShoji, AtsushiHahn, NathanShuba, Mikhail V.Hahn, RainerShukla, GeetanjaliHaider, BilalShvets, PetrHajduk, BarbaraSibag, MarkHaji Sarbatly, RosalamSiddiqui, Irfanul HaqueHaleem, AbdulSiebenhofer, Matthäus S.Hamawand, IhsanSierra-Campos, ErickHameed, Muhammad UsmanSignore, GiovanniHamon, MorganSigusch, Bernd W.Han, KeSilva, ReneHan, XiaojunSilva-Filho, Edson C.Han, YangSilvers, RobertHan, YiyuanSilvestre, WendelHanashima, ShinyaSim, Lee NuangHanifah, Mohamad Fahrul RadziSimion, CristianHansen, Frederik A.Simoes, SandraHansen, UweSingh, Ajaya KumarHao, RunlongSingh, Pramod K.Hao, ZisuSingh, RahulHaponiuk, JózefSingh, Rajesh KumarHappel, ChristineSingh, Rajkumar SunilHaque, Abu Naser Md AhsanulSingh, RandeepHara, NobuoSingh, SatyendraHarja, MariaSingh, VirenderHarnkarnsujarit, NathdanaiSingha, Nayan RanjanHartmann, Anna-MariaSinha, SoumenduHasan Syed, NaveedulSirkar, KamaleshHasbullah, HasrinahSlade, NedaHashaikeh, RaedSławiński, DanielHashem, TawheedSmułek, WojciechHashim, KhalidSnowden, TimothyHashimoto, TakashiSoares, HelenaHassan, AhmedSobierajska, KatarzynaHassan, HassanSohail, MohammadHassan, MohammadSokolova, Maria P.Hassanzadeh-Aghdam, Mohammad KazemSoler, JaimeHassouna, Mohamed Salah El-DinSol-Foulon, NathalieHatami, MehdiSołowski, GawełHawari, Alaa AlSong, WenliangHayashi, TakuyaSong, Young EunHazarika, SwapnaliSosnick, TobinHe, MaoshuaiSperanza, GiorgioHe, MengSpiridon, IulianaHe, QingyaoSpoială, AngelaHe, ShaojianSquintani, Giovanna MaddalenaHe, XuezhongSreekanth Reddy, ObireddyHe, YiStamatin, IoanHeldt, Caryn L.Staszak, KatarzynaHeller, William T.Stathopulos, PeterHellmann, NadjaStehle, YijingHengl, NicolasStelmach, EmiliaHenriquez, FionaStoica, IulianaHeredia Barbero, AlejandroStojmenović, MarijaHernández, AgustínStolarczyk, AgnieszkaHernández, AntonioStopic, SreckoHernandez, SebastianStorr, MarkusHigashi, TomohiroStrandbakke, RagnarHiggs, Paul G.Strub, MatthewHilarydoss, SharonStrzelewicz, AnnaHiramatsu, ToshikiSu, JincaiHirashima, NaohideSu, PeifengHiriart, MarciaSuen, Shing-YiHlaibi, MiloudiSuetsugu, ShiroHo, JamesSuga, KeishiHoldsworth, AlistairSuherman, AlexHolweger, Walter MartinSuhito, Intan RosalinaHong, LiangSui, ShuoHorn, HaraldSukegawa, ShintaroHorzum, NesrinSultan, AbbasHosseini, Seyed SaeidSun, ChengzhenHosseini-Bandegharaei, AhmadSun, ChuanyuHosseinzadeh, MajidSun, HuiHosur, VishnuSun, JieHou, DeyinSun, JunyiHryniewicz, TadeuszSun, PengfeiHu, ChengzhiSun, RuiHu, JunhuaSun, ShuchengHu, LeiqingSun, ShuyuHu, WanheSun, Wei-HsinHu, YangSun, ZhiqiangHuang, ChaoboSunarso, JakaHuang, GuoshengSundarrajan, SubramanianHuang, LiangŠuput, DanijelaHuang, Shu-HsienSýkora, JanHuang, WenyaoSzabó, TamásHuang, YuxiSzczygiełda, MateuszHuet, EricSzekely, GyorgyHühne, RubenSzeleszczuk, ŁukaszHumelnicu, DoinaSzentandrássy, NorbertHung, Yung-TseSzwast, MaciejHussain, AmjadSzymczyk, AnthonyHussain, SameerTabani, Hadi TabaniHwang, Jun YoungTabarkiewicz, JacekHwang, YuhoonTabraiz, ShamasHyun, Dong ChoonTacer Caba, ZeynepIaccino, EnricoTaghizadeh, AliIbba, RobertaTagliaro, IreneIborra-Clar, Maria IsabelTahoon, Mohamed A.Ibrahim, IskanderTaiwo, Abiola EzekielIddya, ArpitaTakamuku, ShogoIdrus, SyazwaniTakeda-Morishita, MarikoIgnjatovic, LjubisaTalluri, Vssl PrasadIhsanullah, IhsanullahTamulevičienė, AstaIkonić, BojanaTan, MingIlia, GheorgeTan, YongzenIliev, BoyanTanabe, YoichiImran, MuhammadTańczyk, MarekInbaraj, Baskaran StephenTang, Bor LuenIngole, Pravin G.Tang, NaInguanta, RosalindaTang, WenzhongIniesta, JesúsTang, XiaobinIonete, Eusebiu IlarianTanifuji, NaokiIqbal, ZafarTaniguchi, TakuyaIrshad, MuneebTapia, Alejandro A.Isakova, ElenaTarditi, Ana MariaIslam, Md AminulTéllez, CarlosIslam, Syed Z.Téllez-Valencia, AlfredoIsmail, ManalTenorio-Alfonso, AdriánIstrate, BogdanTeplyakov, VladimirItagaki, ToruTerashi, GenkiItel, FabianTerenzio Gizzi, FabrizioIulianelli, AdolfoTerzyiska, MargaritaIvanets, AndreiTesi, LorenzoJafarinejad, ShahryarTezel, Fatma HandanJakšić, ZoranThakur, AbhimanyuJamalludin, Mohd RiduanThamaraiselvan, ChidambaramJamnongkan, TongsaiThangarasu, SadhasivamJančić Heinemann, RadmilaThibault, JulesJancikova, SimonaThirumurugan, ArunJang, JaewonThybring, Emil EngelundJansen, Johannes CarolusTian, MiaoJapip, SusiloTichoniuk, MariuszJastrzab, RenataTimofeev, Oleg N.Jáuregui-Haza, Ulises JavierTimsina, RajuJaworska, EwaTiwari, Arjun PrasadJayachandran, MuthuvelTiwari, Rakesh K.Jayakumar, ArunkumarTiwikrama, Ardila HayuJeng, Chung-JiuanToczylowska-Maminska, RenataJeong, SanghyunTodinova, SvetlaJheng, Li-ChengTodorov, SvetoslavJiang, ChenxiaoToikka, AlexanderJiang, GuangdeToko, KiyoshiJiang, JiuxingTomczak, WirginiaJiang, QianqianTomuleasa, CiprianJiang, ShaohuaTonggu, LigeJiang, ZhenTorices, Marcos FallanzaJilani, AsimTorrent-Burgues, JuanJin, HuanyuTorres Rodríguez, MiguelJin, PengruiToth, Andras JozsefJohnson, Colin P.Totu, EugeniaJones, ArwynTrajkovska Petkoska, AnkaJovanović, ZoranTriolo, ClaudiaJullok, NoraTripathi, AmitJunaedi, CholidTrofimova, Marina S.Junaidi, Mohd Usman MohdTrombik, TomaszJurado, AmáliaTrucillo, PaoloKabsch-Korbutowicz, MałgorzataTseng, Hui-hsinKacprzyńska-Gołacka, JoannaTsotsis, Theodore T.Kagramanov, GeorgiyTsui, To-HungKajikawa, TetsuhiroTsunoda, MakotoKalas, WojciechTufa, Ramato AshuKalia, RaghavTuraga, Ravi ChakraKalíková, KvětaTurczyn, RomanKalla, SaritaTurek, MarianKalluru, PolirajuTwu, Ruey-ChingKamali, SaeedUeda, TakumiKanatani, YasuhiroUeda, TaroKanda, KazuhiroUemiya, ShigeyukiKaneko, ToshihiroUlbricht, MathiasKang, Moon-sungUllah, AsmatKannan, KarthikUllah, ZakirKappert, EmielUnterhuber, MatthiasKar, Rajiv K.Upadhyaya, LakshmeeshaKarabelas, Anastasios J.Urbanelli, LorenaKarahan, H. EnisUrbanowska, AgnieszkaKarakoulia, StamatiaUsman, MuhammadKaren, PavelUzdenova, AminatKarimi, MohsenVáclavíková, NatáliaKariper, AfşinVaferi, BehzadKarkhanehchi, HamedVakros, JohnKarl, MatthiasVale, NunoKarn, Santosh Kr.Van Den Bergh, Walter M.Karnjanakom, SurachaiVan Der Bruggen, BartKarthully Soman, SmijinVan Oost, GuidoKaruppasamy, K.Vanangamudi, AnbharasiKassal, PetarVancea, CosminKato, KentaroVandezande, PieterKaur, SukhbirVangala, Janakiram R.Kaushik, GarimaVanneste, JohanKawsar, Sarkar M. A.Varga, ZoltánKazakevicius, EdvardasVaseashta, AshokKazmi, JamalVasos, PaulKececi, KaanVázquez-Ibar, José LuisKepczynski, MariuszVedrtnam, AjitanshuKeser, TomaVega, AlejandroKeshavarz Moraveji, MostafaVelasquez, EliezerKeun-hwan, OhVelizarov, SvetlozarKeusgen, MichaelVenanzi, MarianoKhan, AdnanVenegas, MariaKhan, ArifVenkateshwarlu, KattaKhan, Asim LaeeqVerlicchi, PaolaKhan, Imtiaz AfzalVerma, Shiv PrakashKhan, MasoodVersari, AndreaKhan, Mohd YusufVicente-Manzanares, MiguelKhan, Muhammad IqbalVieira, Roniérik PioliKhan, Muhammad MuzamilVieira, Sandra I.Khan, Muhammad ShujaVigneswari, SevakumaranKhan, Salah Ud-DinVillalain, JoséKhan, Salman AhmadVillarreal-Gómez, Luis JesúsKharissova, Oxana V.Vilsinski, Bruno HenriqueKharraz, JehadVinogradov, MarkelKhataee, AmirrezaViswanathan, BalasubramanianKhitab, AnwarVo, Huyen ThanhKhmelnitskiy, IvanVo, Thuan NgocKhouya, AhmedVögele, MartinKhunová, VieraVolkov, VladimirKhurshid, MohsinVolynsky, PavelKiefer, RudolfVujancevic, Jelena D.Kightlinger, WestonWagh, PriyeshKikhavani, TavanWan Mohtar, Wan Abd Al-Qadar ImadKim, Ae-rhanWang, Chih-FengKim, Beom-junWang, Chih-LiangKim, BumjooWang, ChongqingKim, Dong HyunWang, ChunruKim, Ik-HwanWang, ConanKim, JedeokWang, HanminKim, JinsikWang, HongshengKim, Jung MinWang, HongtaoKim, JungbinWang, HuiyaoKim, Tae KonWang, JianqiangKim, Tae-HyunWang, JinliKimura, NobuyukiWang, JunqiKinoshita, MasanaoWang, QiKirichenko, KseniaWang, QingKirsanov, DmitryWang, RuoxingKjaergaard, MagnusWang, TianyuKlare, Johann P.Wang, TingKlaysom, ChalidaWang, TonghuaKlebert, SzilviaWang, WeiKlingner, AnkeWang, WeihongKobayashi, JunWang, XiaoyuKobrak, Mark N.Wang, XinboKodal, MehmetWang, XueliangKoivisto, AriWang, YaomingKoivula, Hanna M.Wang, YenzenKondo, HiromuWang, YimingKong, LingxueWang, ZhihuaKononova, Svetlana V.Wang, ZhiweiKontogiannopoulos, KonstantinosWang, ZhongzhenKoptyug, AndreyWang, ZiluKorchowiec, BeataWase, NishikantKorneev, DenisWatsa, KhongnakornKorolkov, IlyaWei, DahuaKorupalli, ChiranjeeviWei, DongyangKostka, LiborWeiner, LevKostoglou, MargaritisWen, YouhaiKostov, GeorgiWhitewoods, ChrisKoter, StanislawWhitfield, Phil.Kotsopoulos, ThomasWibisono, YusufKot-Wasik, AgataWidiastuti, NurulKotyńska, JoannaWidomska, JustynaKourkoumelis, NikolaosWiedemann, DominikKoutsioumpas, AlexandrosWieszczycka, KarolinaKovács, EndreWłodarczyk, RenataKowalik-Klimczak, AnnaWnętrzak, AnitaKowalska, IzabelaWojciechowska, MonikaKoyappayil, AneeshWong, Keng YinnKozaderova, OlgaWong, Wai YinKozlov, SergeyWongchoosuk, ChatchawalKraglund, Mikkel RykærWu, BoKramar, PeterWu, KeKrasowska, MonikaWu, QingyunKravchenko, IrynaWu, XingKreutzberger, Alex J. B.Wu, ZhuoxunKrishna Darbha, GopalaXi, LinKrishnamoorthy, SivashankarXia, LinglingKříženecká, SylvieXia, YufeiKruczek, BoguslawXiao, LangqiuKrzan, MarcelXie, LinhuaKubiak-Ossowska, KarinaXu, ChunyanKuhn, RamonaXu, GuangjingKujawski, WojciechXu, KeKula, SławomirXu, PingKulay, LuizXu, RuijieKulbacka, JulitaXu, XiaominKulvelis, Yu V.Xu, XijunKumamoto, EiichiXu, XinKumar, AkshayXu, XuesongKumar, ManuXu, YongKumar, NaveenXuan, ShouhuKumar, PradeepXue, ShuangmeiKumar, RachanaYadav, AnshulKumar, SushilYadav, SudeshKumar, VibhorYam, Kah MengKumar, Yedluri AnilYamashita, Akihiro C.Kumashiro, KristinYan, HaiyangKumawat, Kailash ChandYan, LitangKung, Chung-WeiYanar, NumanKunsági-Máté, SándorYang, BingKuparinen, KatjaYang, Chia-MinKurenkova, AnnaYang, DaijunKurkuri, MahaveerYang, DaliKusumbe, AnjaliYang, FanKuzhir, Polina P.Yang, HsiharngKuzminova, AnnaYang, KesongKuznetsov, AlekseyYang, MengyingKviesis, JorensYang, NiKvitek, OndrejYang, WeiKwak, GijungYang, WenxiuKwon, ByungsuYang, XiaobinKwon, Tae-YubYang, YaweiKyrychenko, AlexanderYang, YingLa Rosa, CarmeloYang, YuLa Rubia, M. DoloresYangali-Quintanilla, Victor AugustoLaezza, FernandaYanilmaz, MeltemLai, Chin WeiYao, ZhichengLai, ZhipingYao, ZhikanLakshminarayana, GandhamYaraki, Mohammad TavakkoliŁamacz, AgataYaremchenko, AlekseyLamm, MeghanYaroslavtsev, AndreyLan, TaoYasukawa, MasahiroLanč, MarekYaxiaer, YalikunLanfredi, SilvaniaYbyraiymkul, DoskhanLang, KeningYeung, VincentLansman, Jeffry B.Yi, MyunggiLarchet, ChristianYi, ZichuanLarin, SergeyYiannourakou, MariannaLarre, IsabelYilmaz, CeyhunLarsen, Andreas HaahrYílmaz, SerkanLarsen, Julie BrogaardYin, YanLasseuguette, ElsaYin, YimingLászló, ZsuzsannaYogarathinam, Lukka ThuyavanLau, Woei JyeYong, Wai FenLäubli, NinoYoo, Dong JinLay, WinsonYoo, Yeong-MinLe, Thien AnYoo, YoungminLebar, Alenka MačEkYoon, Bo KyeongLebensztejn, Dariusz M.Yoon, YisangLecaros, Rumwald LeoYoshida, EriLe-Deygen, Irina M.Yoshida, KazunariLee, AlbertYoshihiko, SanoLee, Chi-YuanYoshimoto, KohkiLee, Chung-HakYoshimune, KazuakiLee, DongkukYounes, HusamLee, DoojinYounis, AdnanLee, Eui-JongYouravong, WiroteLee, IlkeunYousefi Amiri, TaherLee, JieunYu, FeiLee, Jong SukYu, JiujiuLee, Jyh-YeuanYu, Tsyr-YanLee, Kueir-RarnYu, Tzyy LeonLee, SanghoYuan, Dong-HaiLee, Won HeeYuan, ZhanhuiLee, YongkukYue, ZhiLei, HongYunus, Mohd Yusri Bin MohdLei, XiaowenYusof, Nor AzahLeidy, ChadYusuf, MohammadLemke, Horst DieterZádor, ErnőLemonnier, LoicZaharia, CatalinLenzi, Giane G.Zaidi, Syed MohammedLeón-Zerpa, Federico A.Zakaria, ZulfirdausLi, ChenghanZaki, ElsayedLi, ChenyuZambare, Rahul SudhakarLi, FengZandi, RoyaLi, GuoliZarrabi, AliLi, HaoZawada, Adam M.Li, JianrongZayed, Mohamed A.Li, JinchaoZdenek, SloukaLi, JinganZdraveva, EmilijaLi, JinxinZeid, Essam Fadl AboLi, JiushengZelek-Molik, AgnieszkaLi, KejiangZelepuga, ElenaLi, LeiZeng, SicongLi, MeijiaZeng, XiangkangLi, MengfeiZeng, ZihuaLi, MiZhang, ChaoLi, NaZhang, DiLi, SongjieZhang, HanminLi, TianZhang, JiaqiaoLi, Tomi T.Zhang, JinweiLi, WanbinZhang, KaiyueLi, XinZhang, LiLi, XuesongZhang, LiqiuLi, YuhaoZhang, MeijiaLi, ZhanZhang, NingLi, ZhipengZhang, ShaolingLiang, CanzengZhang, TaoLiang, ShuaiZhang, WeiLiang, Yong YeowZhang, WenLiao, BaoqiangZhang, XiaoyuanLiao, Chien-ChangZhang, XihuiLiao, Wei-ChingZhang, XinfangLiao, ZhipengZhang, XuLigorio, CosimoZhang, YaleiLiguori, SimonaZhang, YangLillepärg, JelenaZhang, YanhaoLima, Filipe De SilvaZhang, YanlongLima, Lisa M.Zhang, YataoLima, Maria Helena MeloZhang, YizhouLin, DongqiangZhang, YouwenLin, HaiqingZhang, YujuanLin, HonghongZhang, YuqianLin, JialingZhang, YutingLin, WeichenZhang, ZhengLin, WeichunZhang, ZhimingLin, WeifengZhang, ZidanLin, XiaomingZhao, BiLin, YakaiZhao, JinbaoLin, YuanZhao, NanaLin, YutingZhao, PanLindstedt, SandraZhao, SikaiLing, IreneZhao, YanLipscomb, GlennZheng, JunjianListenberger, LauraZheng, FanLiu, BoZheng, GaofengLiu, JianboZheng, JingxuLiu, JiangtaoZheng, LibingLiu, JianqiangZheng, WeiguangLiu, JunyiZheng, WenweiLiu, RuilinZhongyun, LiuLiu, ShengwenZhou, XuLiu, TaoZhou, YongLiu, TongbaoZhu, QinggongLiu, YangZhu, XuefengLiu, YanlingZieliñska, MagdalenaLiu, YaoxingZienert, TiloLiu, Yen-LiangZille, AndreaLiu, YuanfaZimbudzi, EdwardLiu, ZengcaiZimmerman, William Bauer JayLiu, ZhaopingZirehpour, AlirezaLock, Serene Sow MunZlatar, MatijaLohwacharin, JenyukZnamirowska, AgataLongo, MariagiuliaZobel-Roos, SteffenLópez Garcia, José ManuelŻołądek, SylwiaLopez-Maldonado, Eduardo A.Zounemat-Kermani, MohammadLópez-Maya, ElenaZunita, MegawatiLópez-Zavala, RicardoZuo, JianLoPresti, PatriziaZuppella, PaolaLotfi, SellaouiZydney, AndrewLouis, HitlerŻyłła, Renata

